# Low-Cost Live Insect Scouting Drone: iDrone Bee

**DOI:** 10.1093/jisesa/ieac036

**Published:** 2022-07-06

**Authors:** Jae Hyeon Ryu, Justin Clements, Jerry Neufeld

**Affiliations:** Interstate Drone League (iDrone), POBOX 50082, Boise, Idaho 83705, USA; University of Idaho, 322 E Front St. Boise, Idaho 83702, USA; University of Idaho, 9603 U of I Ln, Parma, Idaho 83660, USA; University of Idaho, 9603 U of I Ln, Parma, Idaho 83660, USA

**Keywords:** *Lygus hesperus*, UAV, integrated pest management

## Abstract

Unmanned aerial vehicles (UAVs, e.g., drones) are a common tool for many civil applications, including precision agriculture, transportation, delivery services, rescue missions, law enforcement, and more. Remote sensing technologies used in conjunction with drones are a dominant application in precision agriculture. Multispectral instrumentation attached to UAVs allows the user to observe multiple parameters, including the normalized difference vegetation index which can represent crop stresses induced by various factors (e.g., drought, insect outbreak, nutrient loss, and other diseases). However, little research has been done to apply drones to accomplish a mission-oriented actionable task in agriculture, such as insect sampling. We propose a low-cost, open source-based live insect scouting drone named ‘iDrone Bee’ to benefit the integrated pest management (IPM) community by minimizing time and efforts of human interventions while collecting live insects in agricultural fields. Herein we present instruction and operation procedures to build and operate an iDrone Bee for insect scouting in an agricultural ecosystem and validate the system in an alfalfa seed field. The findings of this investigation demonstrate that a drone-based insect scouting method may be a valuable tool to benefit the IPM community.

Climate variability and extremes in weather continue to threaten the sustainability of agroecosystems around the world. Increasing temperature and changes in the timing and intensity of precipitation are likely to alter microclimate and local hydrological cycles (Kim and Ryu 2018). Consequently, global warming associated with climate change will continue to effect insect communities and has resulted in increased insect outbreaks and less robust predictive models of phytophagous insect pests (LeHmann 2020). One of the first steps for implementing a robust integrated pest management (IPM) plan in production agriculture is insect scouting, which is used to identify problematic species and inform growers of the timing of pesticide applications. (Foster and Flood 2005). Growers and IPM scouts monitor agricultural fields weekly as soon as crops have emerged from the soil in order to generate metrics for insect thresholds (Foster and Flood 2005). Insect counts can be conducted using a variety of methods, including stand counts, baited insect traps, and field sweeps (Foster and Flood 2005). One of the most robust methods to observe insect pressure within an agricultural field is using a sweep net (Musser et al. 2007). For example, a sweep net with a 38 cm (equivalently 15 in.) diameter is used to take five, 180-degree sweeps in four to five different areas over the alfalfa seed plant fields. The content of the sweep net is then placed within a plastic bag or jar and insect counts are conducted (Neufeld et al. 2019). The western tarnished plant bug (e.g., *Lygus hesperus* Knight, *Lygus lineolaris*, and *Lygus elisus*), are the most damaging insects of alfalfa seed (*Medicago sativa*) in western farmlands in the United States (Strong 1970). Conventionally, growers will scout fields with a standard sweep net and, once the economic threshold of three *L. hesperus* per sweep is reached, growers will make their first application of an insecticide (Walsh 2021). Note that earlier studies for developing the thresholds also suggest variable thresholds through the season (Mayer and Johnsen 1991). This scouting method involves labor-intensive work that is time-consuming, especially as growers are monitoring multiple fields weekly. 

Recently, unmanned aerial vehicles (UAVs) based insect scouting methods have been introduced into agricultural communities where their utility in precision agriculture can be used to monitor insect populations. Park et al. (2018) developed a rotary-wing UAV to monitor insect populations in rice fields with two sweep nets fixed to the legs of the UAV. Neufeld et al. (2019) examined insect scouting using a sweep net equipped with two different drone platforms and compared three different scouting methods, including the standard sweep net method, small drone-based method (P4P), and large drone-based method (M600) to investigate how drone-based scouting methods could be used to assist in scouting alfalfa seed fields. Based on research activities during two consecutive years (2018 and 2019), they demonstrated that drone-based scouting methods can capture *L. hesperus* within an alfalfa seed field. Neufeld et al. demonstrated a high correlation coefficient (0.87) between manual sweep net captures (five 180° sweeps) and a DJI Matrice 600 (M600) with a 15 in. sweep net sweeping a 30 m section of the same field. The result demonstrated that the drone-based scouting method could complement current IPM methods by assessing *L. hesperus* stand counts which can then be used in integrated pest management decisions (Neufeld et al. 2019). Furthermore, Locken et al. (2020) proved that drone-netting (drone with a net attached by rope) is a versatile method for general insect sampling, particularly in inaccessible terrains. Both studies used an off-the-shelf, low-cost, and light-weight quadcopter drone, such as a DJI Phantom 4 Professional (P4P) and a DJI Mavic Pro Platinum (Locken et al. 2020, Neufeld et al. 2019). Neufeld et al. (2019) also incorporated the use of an M600, an industrial drone manufactured by DJI (2020).

However, both previous studies (Neufeld et al. 2019, Locken et al. 2020) suggest that additional investigations introducing automated flight patterns are needed to utilize the advanced drone technology for broader impacts to benefit insect research communities.

Currently, there is no standard protocol to collect insect samples using drones. Additionally, for targeted nondomestic made drones, the ‘Drone Ban’ issued by the U.S. Department of Interior (DOI 2020) and other federal regulations and safety guidelines set by Federal Aviation Administration (FAA) are another challenge to overcome for broader applications in sustainable agriculture. Under the current circumstances, we propose a drone prototype that is low-cost and open source-based for insect scouting named ‘iDrone Bee’ to benefit the IPM communities.

## Materials and Methods

Supplies and materials to produce the iDrone Bee can be purchased from online stores (e.g., Amazon and/or local hardware stores, such as Home Depot) and are listed in Table 1. 

**Table 1.** List of supplies and materials used for iDrone Bee

Category	Components	Number	Cost per unit – currency USD	Total cost – currency USD	Source of materials	Links
Drone architectural frame and parts	Hexacopter Frame	1	$189.98	$189.98	Online store	https://amz.run/5I1t
	30A ESC UBEC	1	$8.49	$8.49	Online store	https://amz.run/5H8x
	620KV Brushless Motors CW/CCW (set of 6)	1	$189.99	$189.99	Online store	https://amz.run/5I2p
	1355 propeller (pair of CW/CCW)	3	$23.99	$71.97	Online store	https://amz.run/5I32
Flight controller module	Raspberry Pi 4 (RP 4)	1	$144.99	$144.99	Online store	https://amz.run/5H8t
	Navio 2	1	$144.99	$144.99	Online store	https://bityl.co/AkWk
Navigation module	GPS/GNSS antenna	1	$12.00	$12.00	Online store	https://bityl.co/AoaO
	Radio Telemetry Kit	1	$28.99	$28.99	Online store	https://amz.run/5HpD https://amz.run/5HpD.
	Taranis Remote Controller (RC)	1	$229.00	$229.00	Online store	https://amz.run/5HqT
	Taranis RC Receiver	1	$39.95	$39.95	Online store	https://amz.run/5HqW
Battery	6s 22.2V Lithium Polymer (LiPo) battery	1	$32.49	$32.49	Online store	https://amz.run/5Hpj
Sweep net attachment	1/16 Stainless Steel Wire^*a*^ (106 m)	1	$24.99	$24.99	Online or local hardware store	https://amz.run/5I20
	Cable Thimble^*a*^ (set of 10)	6	$4.50	$27.00	Online or local hardware store	https://amz.run/5I1y
	Cable hook carabiner^*a*^ (set of 6)	1	$17.84	$17.84	Online or local hardware store	https://amz.run/5I21
	Swivel snap clip^*a*^ (set of 30)	1	$24.99	$24.99	Online or local hardware store	https://amz.run/5I23
	15-inch diameter sweep net	1	$42.39	$42.39	Online or local hardware store or custom build	https://amz.run/5I24

^*a*^Multiple pieces so the price can be reduced with the right number of pieces for the proposed platform.

### Building Instruction

#### Drone Assembly

**Fig. 1.**
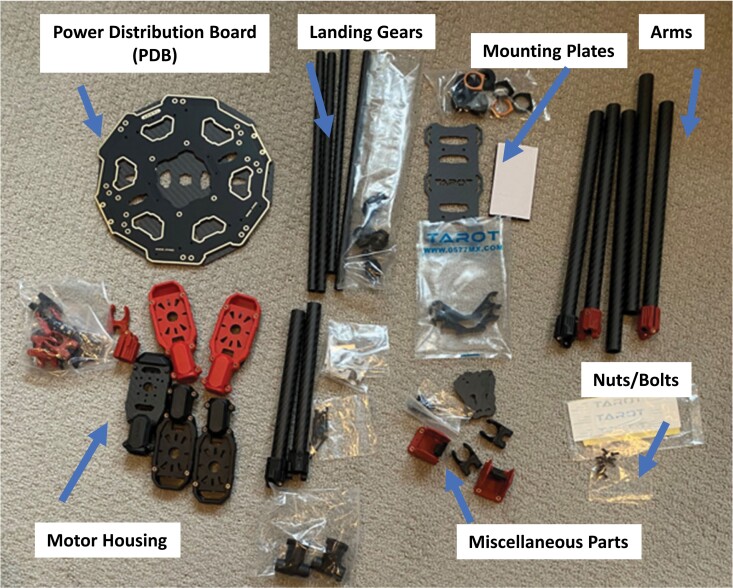
Overview of the hexacopter drone parts.

**Fig. 2.**
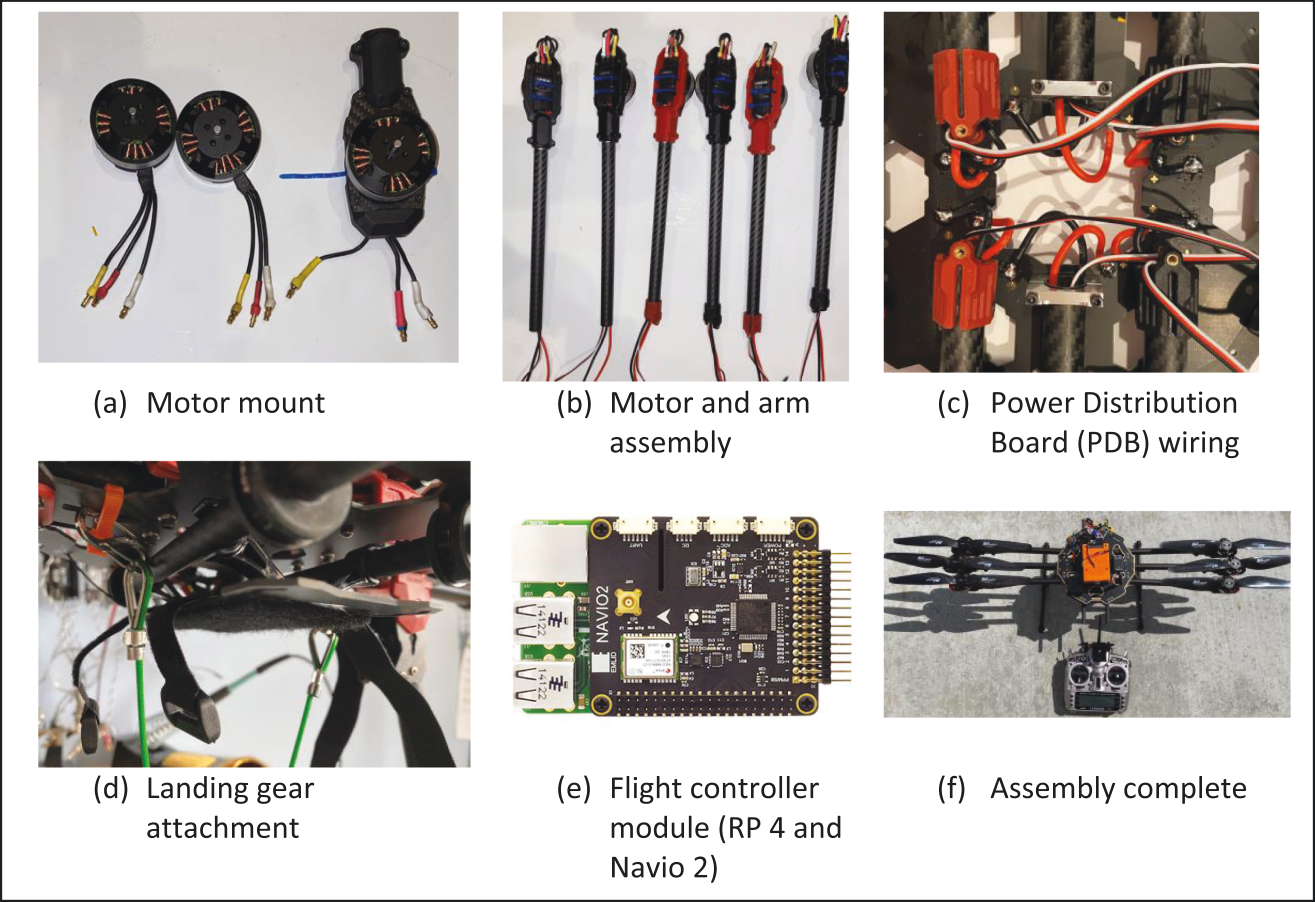
Assembly procedure (a)–(f) to build iDrone Bee.

If the concerns associated with drone restrictions addressed above are not an issue, the readers can skip this building instruction section and go to the sweep net assembly described in the next section. Although minimal soldering efforts are needed to build a hexacopter drone from scratch, the assembly procedure is relatively straightforward using tools (e.g., a set of screw drivers and Alan keys of suitable sizes). First, assemble the body of the hexacopter using necessary parts, including power distribution boards (PDB), arms, landing gears, and mount plats as shown in Fig. 1. The hexacopter was chosen because the operator can maintain flight status for safe landing in case of an emergency associated with single motor failure. Its flexibility and suitability along with simple symmetric design allows the remote pilot to stabilize the drone easily in the air. Three pairs of 620 KV motors—3 clockwise (CW) and 3 counterclockwise (CCW)—are first mounted on the motor housing followed by its connection to arms (or boom) through electronic speed control (ESC) wiring (Fig. 2). Next, the motor and arm assembly shown in Fig. 2b should be attached to the power distribution board (PDB) by soldering all necessary wires, including ESC cables and power cables for the flight controller and batteries. Once the wiring job is complete, landing gears are attached to the hexacopter to finish the assembly. Finally, the flight controller module mainly composed of Raspberry Pi (Raspberry Pi 2022) and Navio 2 (Emlid 2022) is situated in the center top of the hexacopter drone along with the navigation module (Fig. 2). Although a different setup for the flight controller module, such as Pixhawk (Pixhawk 2022) or other affordable options (NAZA 2022) is available, a combination of RP 4 and Navio 2 was used because of its flexibility to accommodate potential sensor-based mission capabilities for further applications (e.g., nocturnal insect scouting). The interested researchers, however, can explore other options when needed.

#### Sweep Net Attachment

**Fig. 3.**
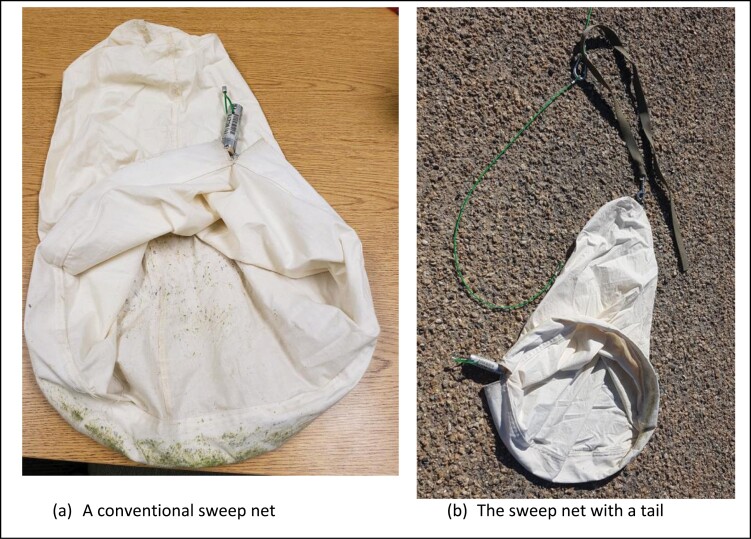
A conventional sweep net with the handle removed.

The 38-cm (15-in.) sweep, which is the same size of a conventional sweep net, is used with the handle removed as shown in Fig. 3. Note that a light weight of a tail attached to the sweep net is also useful to better orient the direction of travel by opening the net and dragging over the alfalfa seed canopy as shown in Fig. 3b. A loop is located at the top of the handle where it is attached to a line coming down from the iDrone Bee. Based on the previous flight test using P4P and M600 (YouTube 2018a, b), it is noted that the rotorwash (downward wind or downdraft) effect should be minimized to avoid the insect flyaway. About 3-m (9.8 ft) wires are suspended from iDrone Bee to minimize downdraft. Additionally, a weight of about 142 g (5 oz) cable hook carabiner was used to avoid tangling wires through flail actions when iDrone Bee is flying back to the launch site with lygus samples. Figure 3 shows a design of the sweep net equipped iDrone Bee.

#### Operations

**Fig. 4.**
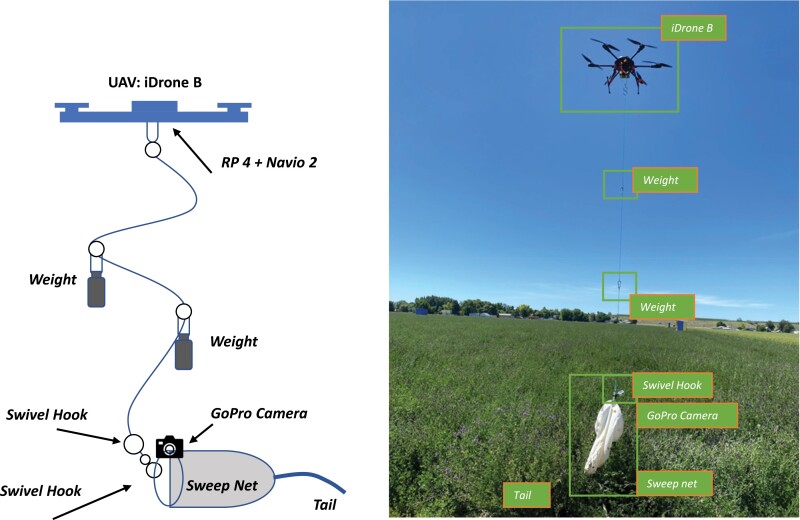
A design of the sweep net-equipped iDrone Bee (left) and its application in the alfalfa seed field (right).

To fly the iDrone Bee in the national airspace, a pilot license (e.g., Part 107) issued by FAA is required (FAA 2022). Unlike a driver license, there is no flying test included to get a remote pilot license for a small unmanned aircraft system (sUAS). The UAS rule may be different in other countries, so it is advised to visit their respective responsible committees and/or agencies for further information. At least two people, including the Pilot-In-Command (PIC) and a visual observer (VO), are needed to fly the iDrone Bee safely; PIC is responsible to control all flight missions while VO can look out to avoid potential air traffic hazards induced by other aerial objects (e.g., crop duster, hawk). Once the sweep net is attached to iDrone Bee securely as shown in Fig. 4, it can take off and fly to collect insect samples. The preflight checklist (see Table 2) must be completed by PIC prior to take off. Although no universal preflight checklist, including failsafe options exists, Table 2 provides useful information for these specific airborne sampling activities using iDrone Bee. As soon as the preflight check is complete by PIC, the iDrone Bee takes off and navigates over the field. The flight path can be determined by PIC with respect to the representation of the desired field that is going to be swept. It is optional, but a low-cost video camera, such as GoPro (GoPro 2022) can be attached to the iDrone Bee to send live streaming during the flight (YouTube 2020c).

**Table 2.** The preflight checklist for iDrone Bee

Category	Preflight^*a*^ status
Weather condition	Is it clear sky to guarantee line-of-sight (LOS) flight mission?
	Is wind speed (less than 8 m/s) adequate for safe flight?
Visual inspection	Have you visually inspected whole system?
	Are all connection wires securely fastened?
	Are six propellers spread well equally to reach the maximum thrust?
Ground control station (GCS)	Is the area clear and leveled for safe take-off and landing?
	Are there flight hazards, such as power lines, building, trees nearby?
	Is the area no drone zone?
	Is there unauthorized personnel nearby?
Operational safety	Are batteries fully charged and secured?
	Are all the switches in the transmitter in the default positions?
	Has the communication between the remote controller and iDrone Bee established?
	Is a safety communication protocol with the visual observer?

^*a*^The preflight checklist must be performed by Pilot-In-Command (PIC).

Additional hardware, such as a video transmitter and receiver, are also needed to display field-of-view (FOV) on ground control station (GCS)’s screen (e.g., Apple iPhone/iPad or Android Phone/Tablet). Once the iDrone Bee approaches the hotspot where insect sampling task is set to take place, the PIC carefully operates and adjusts the direction of travel by dragging the net about 25 cm (10 in.) into the alfalfa seed canopy. The PIC can determine a flight path randomly, but it is recommended to fly iDrone Bee in a linear pattern (e.g., vertical grid or horizontal grid). The procedure of the iDrone Bee takes about 3–5 min to collect insect samples while flying, but it depends on applications. Once the sweep net connected to the iDrone Bee has collected the insects, the PIC can bring it back to the takeoff location. A demo flight is presented in the YouTube Video (YouTube 2020a).

#### Validation

**Fig. 5.**
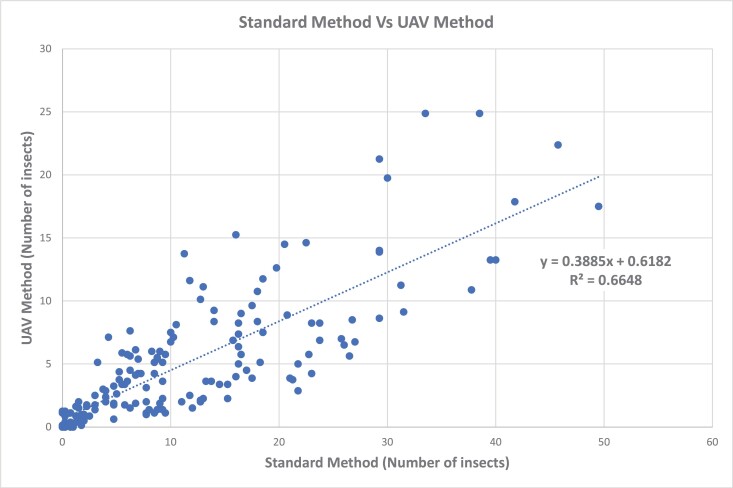
The correlation coefficient (CC) by comparing the number of insect samples captured by the standard method and the UAV method during the 2020 growing season.

The iDrone Bee was field validated in 2020 within three alfalfa seed fields located in the Treasure Valley of Idaho. 168 Drone flights and manual sweeps were carried out to compute a correlation coefficient between the iDrone Bee method and the standard sweep net method (Fig. 5). Briefly, using standard methodology established by Neufeld et al. 2019, we conducted 5 manual sweeps 25 m from the edge of the field using a 38 cm diameter sweep net with 180° sweeps. Once the 5 sweeps were taken, the sweep net was closed, and the content of the bag was transferred to jar containing 95% ethanol. Within the same field locations, the iDrone Bee was used to sweep for *L. hesperus*. The iDrone Bee took off and was positioned aloft. It then started flying at 20 km/h (speed) for 30 m (distance was marked within the field) in a linear pattern (e.g., vertical or horizontal direction). Speed and distance were further monitored with a hand-held GCS mobile app. The iDrone Bee returned to the operator where the contents of the bag were transferred to a jar containing 95% ethanol. *L. hesperus* counts were conducted on jars and correlation coefficient (*R* = 0.66) was generated (Fig. 5).

## Summary and Future Work

Due to technological advances, UAVs (e.g., drones) are commonly used in civil operations, including precision agriculture, transportation, real estate, law enforcement, and delivery services. Although remote sensing research using drones is currently the most common application, drones have demonstrated utility for mission-oriented actionable tasks, such as insect sampling (Neufeld et al. 2019, Locken et al. 2020). More recently, results show that drone-based insect sampling would be an alternative method to collect insect samples by minimizing potential risks (e.g., bee attack or snake bite) associated with intensive labor and personal safety. Additionally, the ‘drone ban’ driven by federal regulations becomes a challenge to overcome for broader application in IPM communities. To resolve this issue, the authors provide instruction on how to build and operate a low-cost insect sampling drone known as ‘iDrone Bee’. Preliminary result shows that the iDrone Bee performs as successfully as the conventional scouting method. Since the iDrone Bee is very versatile, it may serve as a supplement to many other conventional agricultural sampling tools, including spore sampling (YouTube 2020b). When using the iDrone Bee in real-world applications, further specific validation studies should be conducted to develop a conversion factor to relate manual sweep counts to drone sweep counts. Additionally, sampling error and statistical variability from real-world field studies should be explored to determine a minimum number of drone sweeps which are required to be statistically equivalent to manual methods for various cropping systems (e.g., alfalfa seed plant, soybean).

In addition to the method and technology discussed above, iDrone Bee can be fitted with autopilot capabilities, as additional add-on features could minimize human mistake to collect insect samples while navigating any potential complex terrain. Currently, there are no existing drone-based sampling protocols in agricultural fields. The development of these protocols can be developed in the future through hands-on workshops, such as the iDrone Program (iDrone 2022) which brings drone technology to interested farmers and/or consultants.

## References

[CIT0001] DJI. 2020. DJI store. https://store.dji.com.

[CIT0002] DOI. 2020. Secretary Bernhardt signs order grounding interior’s drone fleet for non-emergency operations. https://www.doi.gov/pressreleases/secretary-bernhardt-signs-order-grounding-interiors-drone-fleet-non-emergency.

[CIT0003] Emlid. 2022. Autopilot HAT for Raspberry Pi powered by ArduPilot and ROS. https://navio2.emlid.com/.

[CIT0004] FAA. 2022. Become a drone pilot. https://www.faa.gov/uas/commercial_operators/become_a_drone_pilot/.

[CIT0005] Foster, R., and B. R. Flood. 2005. Vegetable insect management. Meister Pub Co, OH.

[CIT0006] GoPro. 2022. Hero 10: GoPro camera. https://gopro.com/en/us/.

[CIT0007] iDrone. 2022. Interstate/Idaho Drone Program. https://www.idroneprogram.org.

[CIT0008] Kim, J., and J. Ryu. 2018. Modeling hydrological and environmental consequences of climate change and urbanization in the Boise River Watershed, Idaho. J. Am. Water Resour. Assoc. 55: 133–153.

[CIT0009] Lehmann, P., T. Ammunet, M. Barton, A. Battisti, S. Eigenbrode, J. U. Jepsen, G. Kalinkat, S. Neuvonen, P. Niemela, J. S. Terblanche, et al. 2020. Complex responses of global insect pests to climate warming. Front. Ecol. Environ. 18: 141–150.

[CIT0010] Locken, H., O. W. Fischer, J. Selz, and M. Boppre. 2020. Drone-netting for sampling live insects. Insect Sci. 20: 1–3. 10.1093/jisesa/ieaa086 PMC7485588 32915969

[CIT0011] NAZA. 2022. NAZA-M V2. https://www.dji.com/naza-m-v2.

[CIT0012] Neufeld, J., J. Ryu, and J. Barbour. 2019. Development of a UAS-based insect scouting method. J NACAA. 12: 1–5.

[CIT0013] Mayer, D. F., and C. Johnsen. 1991. Integrated insect control practices. *In* Alfalfa seed production and pest management, Western Regional Extension Publ., 12.

[CIT0014] Musser, F., S. Stewart, R. Bagwell, G. Lorenz, A. Catchot, E. Burris, D. Cook, J. Robbins, J. Greene, G. Studebaker, et al. 2007. Comparison of direct and indirect sampling methods for tarnished plant bug (Hemiptera: Miridae) in flowering cotton. J. Econ. Entomol. 100: 1916–1923. 18232411 10.1603/0022-0493(2007)100[1916:codais]2.0.co;2

[CIT0015] Park, H. G., J.-S. Park, and D. -H. Lee. 2018. Potential of unmanned aerial sampling for monitoring insect populations in rice fields. Fla. Entomol. 101: 330–334. 10.1653/024.101.0229

[CIT0016] Pixhawk. 2022. The open standards for drone hardware. https://pixhawk.org/.

[CIT0017] Raspberry Pi. 2022. Raspberry Pi 4. https://www.raspberrypi.com/products/raspberry-pi-4-model-b/.

[CIT0018] Strong, F. E. 1970. Physiology of injury caused by *Lygus hesperus*. J. Econ. Entomol. 63: 808–814.

[CIT0019] Walsh, D. 2021. Pacific northwest plant insect management handbook. Oregon State University, Corvallis, OR, also available at: https://pnwhandbooks.org/sites/pnwhandbooks/files/insect/chapterpdf/legume-grass-field-seed.pdf, accessed on April 18, 2022.

[CIT0020] YouTube. 2018a. Insect collecting by UI team. https://www.youtube.com/watch?v=ODJ_ra1Y0Q0.

[CIT0021] YouTube. 2018b. M600 insect sampling by University of Idaho iDrone team. https://www.youtube.com/watch?v=URHX5G-A4ng.

[CIT0022] YouTube. 2020a. The iDrone B(ee), a insect sampling drone. https://www.youtube.com/watch?v=PzA2V8kshL8.

[CIT0023] YouTube. 2020b. M600 spore side flight. https://www.youtube.com/watch?v=TriW0yP0MK4.

[CIT0024] YouTube. 2020c. The iDrone B(ee) with GoPro is in action during the 2020 growing season. https://www.youtube.com/watch?v=UbjOqAhM8wo.

